# Using high-sensitivity sequencing for the detection of mutations in BTK and PLCγ2 genes in cellular and cell-free DNA and correlation with progression in patients treated with BTK inhibitors

**DOI:** 10.18632/oncotarget.15316

**Published:** 2017-02-13

**Authors:** Adam Albitar, Wanlong Ma, Ivan DeDios, Jeffrey Estella, Inhye Ahn, Mohammed Farooqui, Adrian Wiestner, Maher Albitar

**Affiliations:** ^1^ NeoGenomics Laboratories, Irvine, CA, USA; ^2^ Medical Oncology Service, National Cancer Institute, Bethesda, MD, USA; ^3^ Hematology Branch, National Heart, Lung, and Blood Institute, Bethesda, MD, USA

**Keywords:** ibrutinib, CLL, resistance, PLCγ2, BTK

## Abstract

Patients with chronic lymphocytic leukemia (CLL) that develop resistance to Bruton tyrosine kinase (BTK) inhibitors are typically positive for mutations in BTK or phospholipase c gamma 2 (PLCγ2). We developed a high sensitivity (HS) assay utilizing wild-type blocking polymerase chain reaction achieved via bridged and locked nucleic acids. We used this high sensitivity assay in combination with Sanger sequencing and next generation sequencing (NGS) and tested cellular DNA and cell-free DNA (cfDNA) from patients with CLL treated with the BTK inhibitor, ibrutinib. We also tested ibrutinib-naïve patients with CLL. HS testing achieved 100x greater sensitivity than Sanger. HS Sanger sequencing was capable of detecting < 1 mutant allele in background of 1000 wild-type alleles (1:1000). Similar sensitivity was achieved with HS NGS. No BTK or PLCγ2 mutations were detected in any of the 44 ibrutinib-naïve CLL patients. We demonstrate that without the HS testing 56% of positive samples would have been missed for BTK and 85% of PLCγ2 would have been missed. With the use of HS, we were able to detect multiple mutant clones in the same sample in 37.5% of patients; most would have been missed without HS testing. We also demonstrate that with HS sequencing, plasma cfDNA is more reliable than cellular DNA in detecting mutations. Our studies indicate that wild-type blocking and HS sequencing is necessary for proper and early detection of BTK or PLCγ2 mutations in monitoring patients treated with BTK inhibitors. Furthermore, cfDNA from plasma is very reliable sample-type for testing.

## INTRODUCTION

Bruton tyrosine kinase (BTK) inhibitors like ibrutinib have demonstrated high clinical response rates and durable remissions in patients with chronic lymphocytic leukemia (CLL) including refractory patients to conventional therapy or patients with tumor protein p53 (TP53) mutations [1–5]. Patients who develop resistance to ibrutinib therapy typically have mutations in either BTK or phospholipase c γ 2 (PLCγ2) [1, 6]. Mutations in BTK at the C481S hotspot alter the BTK binding site rendering it reversible to binding ibrutinib which results in a loss of therapeutic activity. Alternatively, mutations in PLCγ2, which is immediately downstream of BTK in the B-Cell receptor signaling pathway, result in a gain of function and BTK independent B-Cell Receptor activation [6–8] While the emergence of these mutations has been reported to be associated with resistance to therapy, little is known about the development of these resistance mutations throughout the course of therapy. In clinical trials of CLL patients on BTK inhibitor (BTKi) therapy, whole exome sequencing with next-generation sequencing (NGS) has typically been used to detect specific mutations in BTK or PLCγ2 genes [1, 6]. Therefore, accurate, high-sensitivity assays that can be run in large volumes in a clinical setting are a necessity to further understand the relationship between the appearance of a mutation and the development of resistance to therapy and clinical progression.

Wild-type blocking polymerase chain reaction (WTB-PCR) followed by Sanger sequencing has demonstrated high sensitivity and versatility in the detection of low frequency mutations [9, 10]. By adding a short (10–12mer) inaccessible [locked or bridged nucleic acid (LNA or BNA)] oligonucleotide, complementary to wild-type hotspot loci, amplification of the wild-type (WT) allele is inhibited, leading to experimentally driven positive selection for mutant alleles. Because a single nucleotide mismatch in the LNA/BNA-DNA hybrid greatly decreases its melting temperature, only mutant template DNA is free to complete its extension. Therefore, WT DNA is amplified linearly but mutant DNA is amplified exponentially [9]. BNA is a third generation nucleic acid analog with excellent mismatch discriminating power and is considered more potent in blocking. Its strong nuclease resistant properties coupled with a 3′ phosphate also prevents amplification of the wild-type DNA and selectively amplifies mutant DNA [11, 12]. The resulting WTB-PCR product can then be sequenced by traditional Sanger sequencing methods. We also theorized that the same principle could be applied to NGS library preparation.

While WTB-PCR/Sanger sequencing or WTB-PCR/NGS can provide accurate, high-sensitivity mutation analysis, spatial sampling bias in patients with lymphomas or CLL with few circulating tumor cells and lymph node or organ involvement could potentially lead to false negatives [13–18]. This is particularly relevant when tumor heterogeneity is considered. The presence of a mutation in a subclone of the tumor cells can be easily missed if the subclone is not circulating or patchy in bone marrow—if bone marrow aspiration is used. In patients with hematologic diseases, the peripheral blood (PB) plasma has been demonstrated to be enriched for tumor-specific DNA, RNA, and proteins [19–22]. This is especially true for the DNA of the more aggressive subclone. Testing cell-free DNA (cfDNA) from plasma or serum may therefore provide greater sensitivity for detecting resistance mutations than cellular DNA from PB.

In this study we describe the development of highly sensitive Sanger and next-generation sequencing strategies for detecting mutations in BTK and PLCγ2 based on WTB-PCR. Using this technology, we demonstrate the development of multiple resistant clones in patients with CLL treated with ibrutinib as they develop resistance to therapy.

## RESULTS

### Significant improvement of sensitivity in detecting BTK and PLCγ2 mutations using HS assay

**Figure 1:**
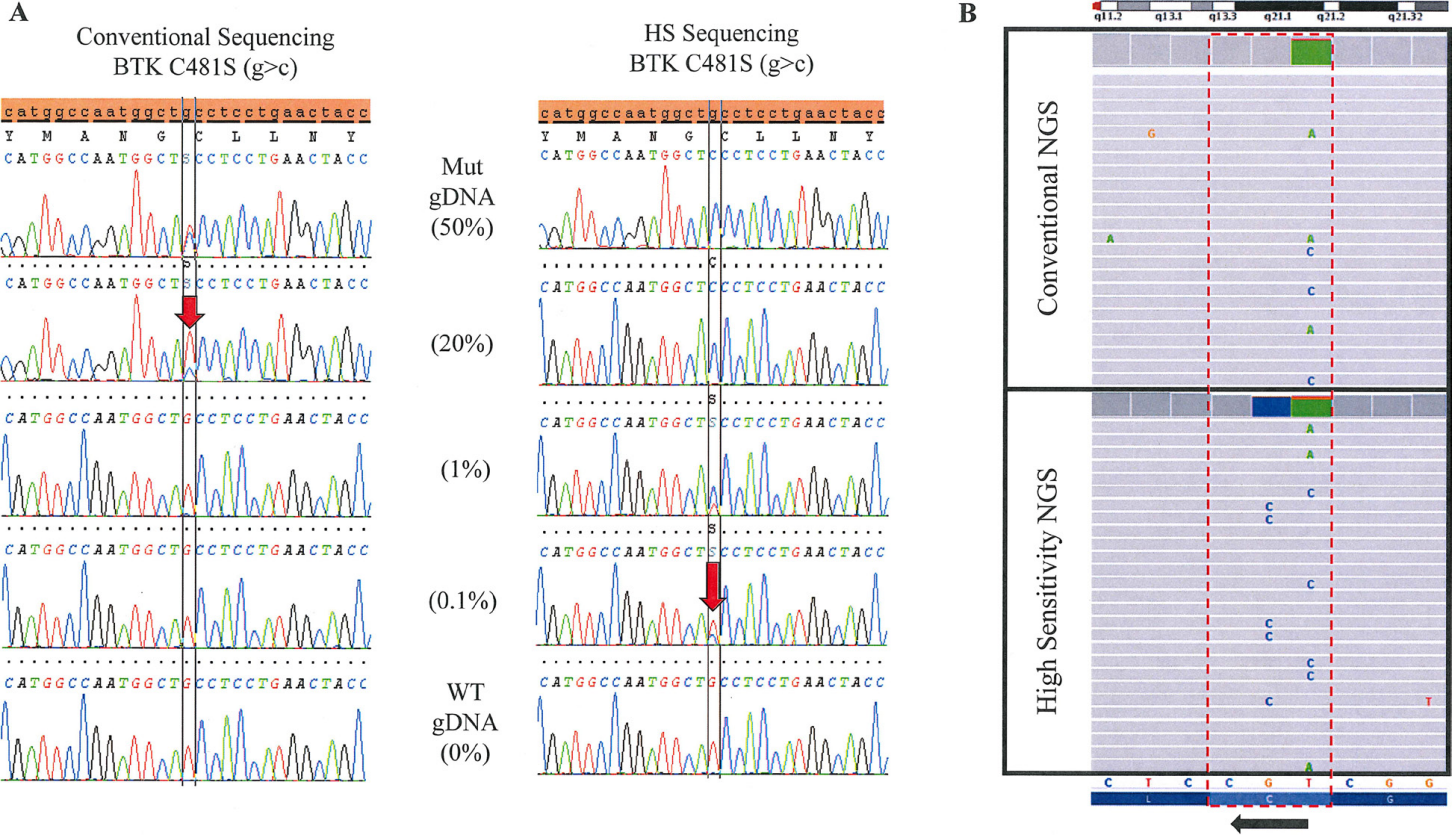
Increase in sensitivity with the addition of BNA/LNA oligonucleotides to sequencing (**A**) Sanger Sequencing. Genomic DNA positive for a BTK C481S (g>c) mutation is serially diluted with WT DNA to determine the limit of detection for the conventional and high-sensitivity (HS) Sanger based assays. Limit of detection is approximately 20% mutant allele in a background of WT by the conventional assay compared with 0.1% in the HS assay. (**B**) Next Generation Sequencing. The lower panel shows the high sensitivity testing of a sample which clearly demonstrates three different DNA strands with three different mutations: c.1442G>C (C481S), c.1441T>C (C481R) and c.1441T>A (C481S). The upper panel shows the same sample tested in conventional NGS without the high sensitivity approach. The three mutations were detectable, but at a lower frequency.

Using the HS assay with WTB-PCR greatly increased sequencing sensitivity when compared to the conventional assay with T-PCR. The conventional assay was able to detect approximately 15–20% mutant allele in a background of WT allele, while WTB-PCR was able to detect as low as 0.1% (Figure 1A). Similar sensitivities were obtained for the PLCγ2 sequencing, however, some loci were more amenable to positive selection by WTB-PCR than others. Sensitivities for exon 19, 20, and 24 of PLCγ2 were 0.2, 1, and 1%, respectively, mutant allele in a background of WT by HS assay (data not shown).

### Lack of mutation in BTK and PLCγ2 in patients with BTKi-naïve CLL

Using HS sequencing we tested samples from 44 patients with newly diagnosed CLL or after therapy with FCR. None of these patients showed mutations in BTK exon 15 or PLCγ2 Exon 19, 20 and 24 genes.

### Mutations in BTK and PLCγ2 after BTKi detected by HS

We tested 63 samples from 16 patients with CLL treated with ibrutinib who were suspected of resistance or who showed evidence of progression while on therapy. Using conventional Sanger sequencing only 21% of tested samples showed mutations in BTK, while the HS testing showed mutations in 43% of tested samples (*P* < 0.00001). Mutations in PLCγ2 were detected in 5% of tested samples using conventional Sanger sequencing and in 33% of samples using HS testing (*P* < 0.00001). The mutations detected included BTK: C481S and C481R; PLCγ2: R665W, L845F, S707Y, P664S, P664L, Ser707TyrdelAlaTyr (6NT deletion). Without HS testing 56% of positive samples would have been missed for BTK (*N* = 27) and 85% of PLCγ2 (*N* = 20) would have been missed. No mutations detected by the conventional assay were missed by the HS assay.

### Multiple subclones with BTK and PLCγ2 mutations in BTKi resistant patients detected using HS sequencing

Overall, of the 16 patients on therapy with ibrutinib and suspected resistance or disease progression, 11 (69%) had a mutation in either BTK or PLCγ2, 6 (37.5%) patients had mutations in both genes, and 2 (12.5%) patients had three or more mutations that were detected by HS assay. By comparison, using conventional assay only 6 (37.5%) patients had mutations in either BTK or PLCγ2, 1 (6.3%) had mutations in both genes, and 1 (6.3%) patient had three or more mutations. More than half of the patients with mutations (55%, *N* = 11) had multiple drug resistant mutations that are detectable by the HS assay and two patients had 5 separate mutations (Table 1). The fact that we were able to see three separate subclones (as determined by NGS; Figure 1B) in at least one patient (Patient # 4) suggests that these other mutations also exist in separate subclones. Without HS testing, 83% of the additional clones would have been missed.

**Table 1:** Tested patients with suspected clinical progression on ibrutinib therapy

	BTK	PLCγ2	BTK HS	PLCγ2 HS	% CLL/ WBC
Pat # 1	WT	WT	WT	R665W (c>t) (cells);P664S (c>t) (plasma)	7.0
Pat # 2	WT	WT	WT	R665W (c>t)	87.8
Pat # 3	C481S (g>c)	WT	C481S (g>c)	R665W (c>t),S707Y (c>a),L845F (a>t, a>c)	61.6
Pat # 4	C481R (t>c)	R665W (c>t),L845F (a>t)	C481S (g>c, t>a),C481R (t>c)	R665W (c>t),L845F (a>t)	55.0
Pat # 5	C481S (g>c)	WT	C481S (g>c)	Ser707TyrdelAlaTyr (6NT deletion)	48.9
Pat # 6	WT	WT	C481S (g>c)	R665W (c>t)	21.5
Pat # 7	C481S (g>c)	WT	C481S (g>c)	Ser707TyrdelAlaTyr(6NT deletion) (serum);S707Y (c>a) (plasma)	75.0
Pat # 8	C481S (g>c)	WT	C481S (g>c)	WT	not performed
Pat # 9	WT	WT	WT	WT	not performed
Pat # 10	WT	WT	WT	WT	not performed
Pat # 11	WT	WT	WT	WT	not performed
Pat # 12	WT	WT	WT	WT	not performed
Pat # 13	WT	WT	WT	WT	not performed
Pat # 14	WT	WT	C481S (g>c)	P664L (c>t)	not performed
Pat # 15	WT	WT	C481S (g>c)	WT	not performed
Pat # 16	C481S (g>c)	WT	C481S (g>c)	WT	92.9

Mutational status of BTK and PLCγ2 was determined by conventional and high-sensitivity (HS) Sanger sequencing. “% CLL/WBC” indicates the percentage of CLL cells of white cell count in the tested samples as determined by flow cytometry. Abbreviation: Pat = Patient, HS = high-sensitivity, WBC = white blood cells, WT = wild-type/unmutated.

Seven of the mutations in PLCγ2 (87.5%, *N* = 8) and three mutations in BTK (33%, *N* = 9) that were detectable by HS assay at progression were undetected by conventional assay. Median percentage of CLL cells in these samples as tested at progression was 58% (*N* = 8, range = 7–93%) as determined by flow cytometry.

### Next-generation sequencing and improvement of sensitivity using blocking oligonucleotides

In general, resistance mutations in BTK or PLCγ2 were detected by NGS in all tested samples, except for two samples: Patient # 5, who had a very low frequency PLCγ2 Exon 20 6NT deletion and patient # 3, who had two low frequency PLCγ2 Exon 19 R665W and Exon 20 S707Y mutations. The addition of BNA/LNA oligonucleotides enriched for BTK and PLCγ2 hotspot mutations (Table 2 and Figure 1B). In addition, NGS showed that when multiple mutations were detected in one sample, these mutations were not in tandem and were therefore present in different strands of DNA (Figure 1B). In particular, a sample from patient # 4, in which three BTK mutations were detected, the three mutations were completely independent events existing in separate DNA strands, thus suggesting different subclones.

**Table 2:** Increased next-generation sequencing sensitivity with the addition of BNA/LNA oligonucleotides

			High-Sensitivity NGS		Conventional NGS	
**Patient # 4**						
Gene	Nucleotide	Amino Acid	Alternate Variant Frequency	Read Depth	Alternate Variant Frequency	Read Depth
PLCγ2	1993C>T	Arg665Trp	16.6	353	9.6	912
PLCγ2	2535A>T	Leu845Phe	62.7	126	14	700
BTK	1442G>C	Cys481Ser	5	309	4	681
BTK	1441T>A	Cys481Ser	6	310	7	680
BTK	1441T>C	Cys481Arg	14.9	307	6	680
Patient # 16						
Gene	Nucleotide	Amino Acid			Alternate Variant Frequency	Read Depth
BTK	1442G>C	Cys481Ser			51.2	697
Patient # 5						
Gene	Nucleotide	Amino Acid	Alternate Variant Frequency	Read Depth		
BTK	1442G>C	Cys481Ser	3	398		
Patient # 15						
Gene	Nucleotide	Amino Acid	Alternate Variant Frequency	Read Depth		
BTK	1442G>C	Cys481Ser	2	89		
Patient # 3						
Gene	Nucleotide	Amino Acid	Alternate Variant Frequency	Read Depth		
BTK	1442G>C	Cys481Ser	73.2	102		
PLCγ2	2535A>T	Leu845Phe	3	145		
PLCγ2	2535A>C	Leu845Phe	5	145		

All samples used were from patients with suspected progression. High-sensitivity NGS includes BNA/LNA oligonucleotides in library preparation and conventional NGS does not.

### Testing using cell-free DNA

We performed parallel HS sequencing of 9 temporally matched pairs of plasma cfDNA and cellular DNA. Of these 9 pairs, 4 parallel cfDNA isolated from serum were also tested. Of the 9 plasma cfDNA samples, 7 (78%) showed mutations in BTK and 4 (44%) showed mutations in PLCγ2. The cellular DNA showed mutations in BTK in 7 (78%) samples, but only 2 (22%) mutations were detected in the PLCγ2 gene. Of the 4 serum cfDNA samples, only 1 (25%) showed a mutation in BTK, and 1 (25%) had a mutation in PLCγ2 (Table 3A).

Table 3Cell-free DNA from peripheral blood plasma is more sensitive than serum and cellular DNA

**Table:** 

A.						
		Serum	Plasma		Cells	
Samples Tested		4	9		9	
BTK Mutation		1 (25%)	7 (78%)		7 (78%)	
PLCγ2 Mutation		1 (25%)	4 (44%)		2 (22%)

**Table:** 

B.								
	BTK					PLCγ2		
	Tested Samples			Mut		Tested Samples		Mut
Cells/Plasma	9/9			7/7 (100%)		9/9		2/4 (50%)
Serum/Plasma	4/4			1/3 (33%)		4/4		1/3 (33%)

(A) High-sensitivity testing of 9 temporally matched plasma and cellular samples from the same patients of which 4 serum samples were also available indicates that plasma may be enriched for tumor specific DNA more so than serum and cells. (B) Comparison of mutation status between paired sample-types.

All mutations detected by either cellular DNA or serum cfDNA were also detected by plasma cfDNA. 2 out of 4 (50%) PLCγ2 mutations identified by plasma cfDNA were not detected by cellular DNA in the total 9 paired samples. 2 out of 3 (67%) of both BTK and PLCγ2 mutations identified by plasma cfDNA were not detected by serum cfDNA in the total 4 paired samples (Table 3B). It is difficult to quantify the mutation percentage using Sanger or WTB-PCR and sequencing, but overall, mutant peak was relatively significantly stronger in plasma samples as compared with cell samples in almost positive cases.

The number of patients is too small and we cannot determine if plasma positivity correlated with more enlarged lymph node or lower number of circulating lymphocytes, since most patients with progression also had enlarged lymph nodes. Some of the patients with detectable mutations in cells also had low percentage of circulating CLL cells.

## DISCUSSION

Given the association of BTK and PLCγ2 mutations with resistance to ibrutinib therapy, an accurate, highly sensitive assay—capable of being run in large volume—is a necessity. Using WTB-PCR with Sanger sequencing or NGS has multiple advantages in the clinical setting. Increased sensitivities of up to 0.1% mutant allele in a background of wild-type (Figure 1A) could allow clinicians to detect the presence of resistance mutations early on during the course of therapy. Knowing early on when mutations conferring resistance to therapy emerge and that the majority of the time they co-develop alongside additional sub-clones with resistance mutations (Table 1) may be very helpful in devising a strategy to overcome evolving resistance by, for example, adding additional therapeutic agents.

WTB-PCR/Sanger or WTB-PCR/NGS testing allows broad coverage of mutation hot-spots and the detection of undiscovered mutations; they also provide adequate internal controls for ruling out false positives. Its additional utility in revealing low frequency mutant populations, especially in plasma cfDNA is invaluable and will guide future research.

Because we know the limit of detection for the conventional assay is approximately 15%, mutations that are detectable by HS assay but not by conventional assay are therefore present in only a small fraction of CLL cells at progression despite the relatively high CLL cellularity in the tested samples (Median = 58%). The low percentage of CLL with the resistance mutations at time of progression implies that these mutations may have secondary effects on CLL cells lacking BTKi resistance mutations perhaps via tumor microenvironment resulting in loss of therapeutic activity [23–25]. We are analyzing peripheral blood samples and there is a possibility that lymph nodes may contain more significant number of cells with mutation. However, testing lymph nodes might not be a practical approach, especially when these lymph nodes are deep and not easily accessable. Furthermore, the pattern of multiclonal BTK inhibitor resistance is unique, perhaps because of the chronic nature of the disease. In more acute malignancies, clonal evolution is typically linear with one subclone outcompeting the others and giving rise to resistance [26, 27]. In these cases, however, we observed that 6 of the 16 patients at progression have multiple, persisting subclones (Table 1).

**Figure 2:**
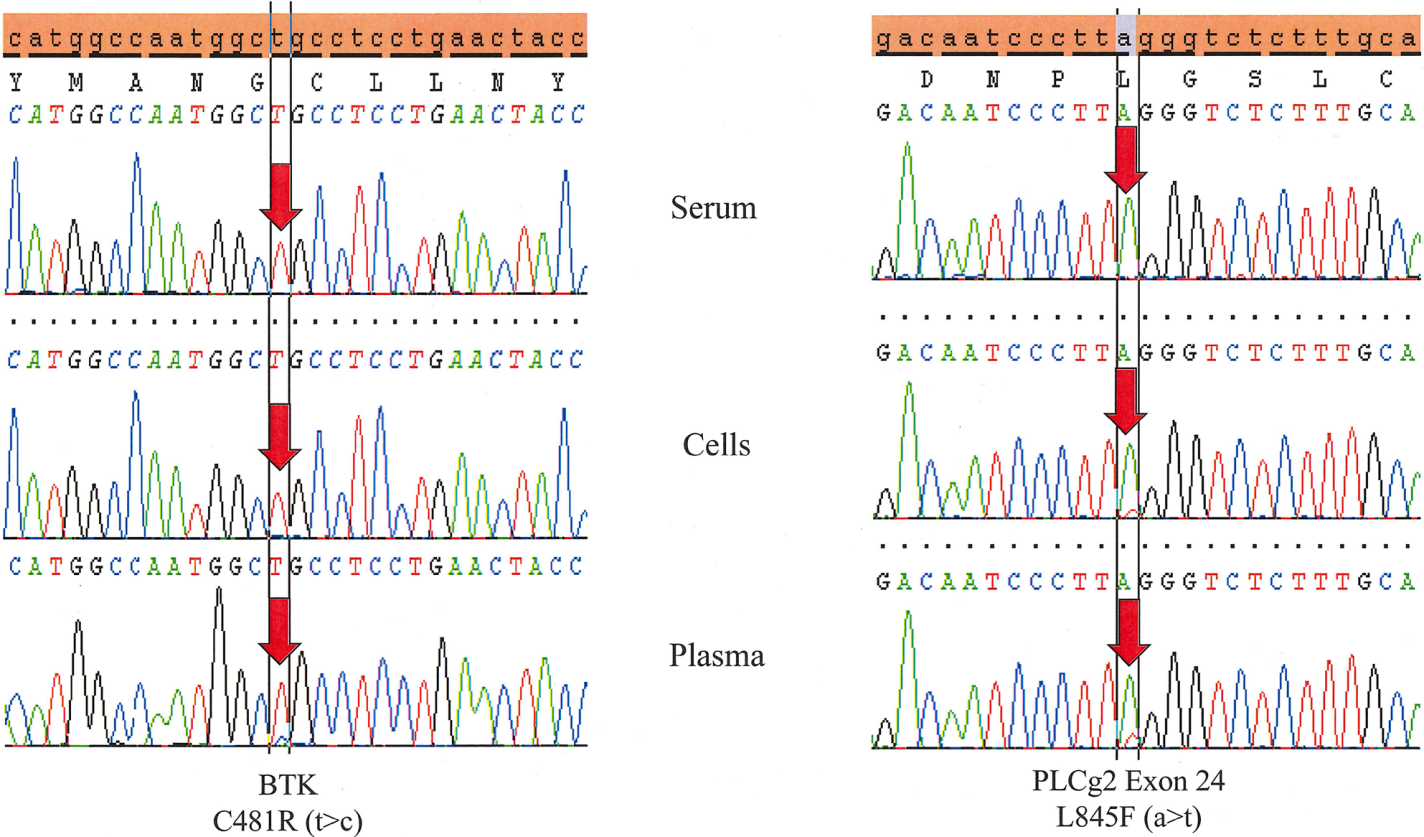
Testing DNA derived from peripheral blood plasma is more sensitive than serum and even cells Temporally matched samples from the same patient (Patient # 4) reveals both a BTK C481R (t>c) and a PLCγ2 L845F (a>t) mutation in plasma. The BTK mutation is not detected in cells and both mutations are absent in serum.

Plasma cfDNA from PB is more sensitive for detecting resistance mutations than cellular DNA or serum cfDNA (Table 3, Figure 2). While no cellular DNA or serum cfDNA samples with mutant BTK or PLCγ2 were wildtype in their respective plasma cfDNA samples, 50% of the PLCγ2 mutations detected in plasma DNA were not detected in their respective cellular DNA samples. Furthermore, 67% of both BTK and PLCγ2 mutations detected in plasma were not detected in serum. Unfortunately no more plasma samples from patients with progression were available for testing. Although more testing of more plasma samples is needed for confirmation, this approach may be very useful in screening patients for resistance mutations, especially in patients with lymphomas or CLL with few circulating tumor cells and lymph node or organ involvement. Because the resistance mutations allow these cells to proliferate despite BTKi therapy, one plausible explanation for this result is that the increased proliferative rate and consequent improper processing of the cellular contents results in plasma enriched with DNA derived from CLL cells possessing resistance mutations. In serum, the coagulation process may unintentionally lyse fragile cells like granulocytes, which has the effect of diluting the serum with non-tumor cfDNA.

In conclusion, our data indicates that incorporating WTB-PCR into Sanger sequencing or NGS is a highly sensitive and invaluable tool in screening and monitoring patients on ibrutinib or other BTKi therapy for resistance mutations. Additionally, plasma from peripheral blood may be more sensitive than serum and even cells in detecting the presence of these resistance mutations. Although the number of cases is small and further confirmation is needed, using these tools we show that multiple low-frequency subclonal populations of CLL with resistance mutations in BTK and PLCγ2 are quite common in patients who progress on therapy with ibrutinib. Using this information we can monitor, with increased accuracy, patients on BTKi therapy and make more informed therapy decisions when we detect the presence of mutations known to result in a loss of therapeutic activity.

## MATERIALS AND METHODS

### Patients and samples

Samples were collected from ibrutinib-naive CLL patients as well as from patients treated with ibrutinib as a part of the single-arm, phase-2 study of single agent ibrutinib in CLL with and without 17p deletions conducted at the NIH (NCT01500733). We tested 44 DNA samples from BTK inhibitor naïve patients with CLL by the high-sensitivity (HS) assay for mutations in BTK exon 15 and PLCγ2 Exon 19, 20, and 24. This included samples from PB, bone marrow aspirate, and fresh lymph node tissue. We also tested 16 patients with CLL that were on ibrutinib therapy and had suspected resistance or disease progression. The clinical characterstics of these patients is described in details in reference [28]. Briefly, progression was defined as ≥ 50% increase in sum of the product of the diameters of representative lymph nodesor ≥ 50% increase in absolute lymphocyte count, confirmed in two consecutive assessments and with an absolute B-cell count > 5,000/uL [28]. These duration of ibrutinib therapy in these patients varied between one and three years [28].

Prior treatment data was available for 15 of the 16 patients. Median time from CLL diagnosis until beginning ibrutinib therapy was 5.1 years (range: 0.6–14.9). 3 patients were treatment naïve and 12 were relapsed/refractory (RR) CLL; median number of prior therapies for RR patients was 3.5 (range: 1–6). From these 16 patients we tracked the emergence of resistance mutations in BTK or PLCγ2 by both the HS and conventional assay using a total of 63 samples collected over a 43-month period. This also included samples from PB cells (*N* = 39), plasma (*N* = 10), serum (*N* = 11), and bone marrow aspirate (*N* = 3). Samples were either de-identified and tested according to IRB-approved protocol or tested after obtaining a consent form.

From these samples we also performed HS sequencing on 9 temporally matched pairs of plasma cfDNA and cellular DNA. Of these 9 pairs, 4 parallel cfDNA samples isolated from serum were also tested.

DNA extraction: We extracted DNA from PB cells, bone marrow aspirate, and fresh tissue using the QIAamp DNA Mini Kit (Qiagen; Venlo, Netherlands) in both manual and automated (QIAcube) extractions according to manufacturer's instruction. Extracted DNA was then quantified using a Nanodrop 2000 (Thermo Fisher Scientific; Waltham, MA, U.S.A.) instrument and adjusted to approximately 50–100 ng/μL with H_2_O.

Total nucleic acid was extracted from PB plasma and serum via the NucliSenS EasyMAG automated platform (BioMerieux; Marcy-l’Étoile, France). DNA was then quantified using Qubit 2.0 Fluorometer (Thermo Fisher Scientific; Waltham, MA, U.S.A.) and adjusted accordingly.

### High-sensitivity and conventional sanger DNA sequencing

The BTK inhibitor resistance assays were developed to amplify exon 15 of BTK and exon 19, 20 and 24 of PLCγ2. 0.25 μL FastStart Taq DNA Polymerase, 5 U/μl in storage and dilution buffer Fast Start Taq DNA polymerase (Roche; Basel, Switzerland), 2.5 μL PCR reaction buffer 10× w/ 20 mM MgCl_2_, 250 μM dNTPs (Invitrogen; Waltham, MA, U.S.A.), 0.4 μM forward primer, 0.4 μM reverse primer (IDT; Coralville, IA, U.S.A.) (Table 4), and 2 μL genomic DNA (50–100 ng/μL) were added to DNAse, RNAse-free, ultra-pure H_2_O to create a final solution volume of 25 μL per reaction. All PCR primers were designed with a 5′-M13 sequence (M13-forward: tgt aaa acg acg gcc agt; M13-reverse: cag gaa aca gct atg acc) to allow for annealing of complementary sequencing primers. The HS assays were identical to their conventional counterparts except for the addition of BNA (Bio-Synthesis; Lewisville, TX, U.S.A.) or LNA (Exiqon; Woburn, MA, U.S.A.) oligonucleotides A3, B3, C3, and D3 (Table 4) being added to the master mixes of BTK and PLCγ2 Exon 19, 20, and 24, respectively. A3 was added to the BTK master-mix at 4 μM; B3 to PLCγ2 Exon 19 at 4 μM; C3 to PLCγ2 Exon 20 at 40 nM; D3 to PLCγ2 Exon 24 at 4 μM. The LNA oligos were designed to feature a 3′ inverted dT to inhibit both extension by DNA polymerase and degradation by 3′ exonuclease. The BNA oligos were designed with a 3′ phosphate for the same reason. All reactions were subjected to identical thermocycler settings; initial denaturation at 95°C for 6 minutes; 40 cycles of denaturation at 95°C for 30 seconds, primer annealing at 56°C for 30 seconds, and extension at 72°C for 1 minute 20 seconds; this was followed by a final extension at 72°C for 10 minutes. PCR products were purified using Agencourt AMPure XP magnetic beads (Beckman Coulter; Brea, CA, U.S.A.), bi-directionally sequenced using a BigDye Terminator v3.1 Cycle sequencing kit (Life Technologies; Waltham, MA, U.S.A.), and subjected to ethanol precipitation. The precipitated DNA was then resuspended in 10 μL Hi-Di Formamide (Life Technologies; Waltham, MA, U.S.A.), denatured at 95°C for 3 minutes, and run on the ABI 3730XL sequencer. Sequencing data were base-called by sequencing software and analyzed by ABI Prism^®^ SeqScape software.

**Table 4:** Primers and BNA/LNA oligonucleotides

	Oligo Name	Sequence
A1	BTK-FW	5′-tgt aaa acg acg gcc agt CAG TTG TAT GGC GTC TGC AC-3′
A2	BTK-REV	5′-cag gaa aca gct atg acc TCC AGG TAT TCC ATG GCT TC-3′
A3	BTK-BNA	5′-G+GA+G+G+C+A+G+C+CAT+TG-[Phosphate]-3′
B1	PLCγ2-Exon19-FW	5′-tgt aaa acg acg gcc agt GCT CAC CTG GTC GTT TTC C-3′
B2	PLCγ2-Exon19-REV	5′-cag gaa aca gct atg acc CAA GCC CCT CTG TAG AGC AT-3′
B3	PLCγ2-Exon19-LNA	5′-+G+A+T+T+C+CC+C+G+G/3InvdT/-3′
C1	PLCγ2-Exon20-FW	5′-tgt aaa acg acg gcc agt AAA AAT TGT TTG GCC ACC AG-3′
C2	PLCγ2-Exon20-REV	5′-cag gaa aca gct atg acc TGG TGA ATA CTC AGA GGT TTG C-3′
C3	PLCγ2-Exon20-BNA	5′-G+G+AC+C+T+C+CG+C+CT-[Phosphate]-3′
D1	PLCγ2-Exon24-FW	5′-tgt aaa acg acg gcc agt AAA CGG TGT GCT TTG GAA AC-3′
D2	PLCγ2-Exon24-REV	5′-cag gaa aca gct atg acc AGA CAG GAC CCT GTG TCA GC-3′
D3	PLCγ2-Exon24-LNA	5′-+C+T+T+A+G+G+G+T+C+TC/3InvdT/-3′

Abbreviation: FW = forward, REV = reverse, +N = BNA/LNA bases.

In order to determine the sensitivity and limit of detection, dilution series experiments with genomic or amplicon DNA were carried out. Genomic DNA taken from samples that tested positive for BTK (C481S) or PLCγ2 (R665W) or amplicon DNA with PLCγ2 (S707Y, L845F) mutations were quantified using a Qubit dsDNA high-sensitivity assay kit (Invitrogen; Waltham, MA, U.S.A.). This DNA was serially diluted with WT DNA of the same type.

### Next-generation DNA sequencing

We applied the WTB-PCR principle to custom SureSelect QXT Target Enrichment (Agilent; La Jolla, CA) and Nextera Rapid Capture (Illumina; San Diego, CA) panels with the addition of the BNA/LNA oligonucleotides (A3, B3, C3, D3; Table 4) in order to increase our limit of detection for the hotspot mutations in hybrid-capture based NGS. A3, B3, and D3 were added to library preparation at a working concentration of 2 μM and C3 was added at 40 nM. Both panels cover 315 genes that include the BTK and PLCγ2 genes. One sample that was positive for resistance mutations in BTK and PLCγ2 was tested by Nextera Rapid Capture based assay with and without WTB-PCR in order to determine if mutant enrichment could be achieved in the NGS setting. One additional sample with resistance mutations was tested by the same assay without WTB-PCR. The SureSelect QXT Target Enrichment based assay with WTB-PCR was used on 3 additional samples with resistance mutations.

## Abbreviations

BTK: Bruton tyrosine kinase, PLCγ2: Phospholipase c gamma 2, cfDNA: Cell-free DNA, HS: High sensitivity, NGS: Next generation sequencing, WTB-PCR: Wild-type blocking-polymerase chain reaction
